# How GPs approach physical activity discussion in routine care: a qualitative study of video-recorded consultations

**DOI:** 10.3399/BJGPO.2024.0253

**Published:** 2025-07-02

**Authors:** Neha Giri, Kimberley Norman, Elizabeth Ann Sturgiss

**Affiliations:** 1 School of Primary and Allied Health Care, Monash University, Melbourne, Australia; 2 Faculty of Health Sciences and Medicine, Bond University, Robina, Australia

**Keywords:** exercise, general practice, primary health care, communication, prevention

## Abstract

**Background:**

Physical activity (PA) engagement is critical for improving health and wellbeing across nearly all patient health concerns. General practitioners (GPs) are well positioned to have discussions about PA owing to the frequency with which they see their patients over long periods of time.

**Aim:**

To explore (a) how PA is discussed and (b) the types of PA that are discussed in real-world GP–patient consultations.

**Design & setting:**

A qualitative descriptive study using real-world video-recorded consultations with Melbourne-based GPs.

**Method:**

Secondary analysis of 43 video consultation data from four GPs held with the Digital Library repository at Monash University, Australia. Two researchers reviewed the consultations and analysed each consultation using descriptive content analysis.

**Results:**

From the total consultations (*n* = 43), 41.9% (*n* = 18) discussed PA. Five consultations included a structured GP management plan that prompted PA questions for discussion. GPs had a patient-centred approach and discussed a range of different types of PA, which was tailored to the specific health needs of each patient. These included exercise prescription, general advice, aerobic exercise, functional movement, and allied healthcare referrals.

**Conclusion:**

GPs discuss PA with patients in opportunistic or systematic ways using a patient-centred approach. This study could be used as a foundation for establishing teaching resources for GPs, both in training and in professional development. Future studies could explore how PA discussions improve health outcomes over longer periods of time. This will contribute to understanding how, and if, PA is followed-up to further understand the effectiveness of these discussions.

## How this fits in

GPs play a key role as an initial primary care contact for patients and hold a responsibility to enquire and recommend physical activity to encourage disease prevention and health maintenance. However, there is limited research about how GPs address physical activity with patients. This study presents data from Australia’s only digital library of real-world video-recorded GP–patient consultations. We used a novel approach to understand how GPs discuss physical activity and the type of physical activity they discuss in consultations. This research establishes a foundation for GP teaching resources and professional development in routine care.

## Introduction

In recent decades, there has been a focus on recognising and promoting the significance of physical activity (PA) in health care. Similar to other countries, including the UK, Canada, and the US,^1–3^ GPs in Australia include the topic of PA engagement with their patients in consultations.^4^ For substantial health benefits the Royal Australian College of General Practitioners (RACGP) recommends that adults engage with moderate or vigorous PA for 1.25–5 hours per week, depending on personal health circumstances.^4^ Aerobic-based PA is the most widely recommended exercise type for all ages as the majority of international PA guidelines, including the World Health Organization (WHO), recommend a target of 150 minutes per week of moderate-intensity aerobic PA.^5^ However, it is of concern that globally, 25% of adults and more than 80% of adolescents do not meet these PA guidelines.^6^

PA plays an important role in optimal health maintenance and the prevention of further health conditions.^7,8^ While the optimum levels of engagement with PA can be different for each patient owing to individual limitations, lifestyle factors, time, and personal worldviews of PA, international guidelines indicate that any form of PA engagement is better than none and is a useful tool to enhance patients’ health and wellbeing. In Australia, GPs often serve as patients’ initial and primary point of healthcare contact.^4,9,10^ Given the longitudinal nature of GP–patient relationships, GPs can provide essential guidance to patients at key times in the life course.^11^ Australian GPs have reported using GP management plans to discuss PA.^12,13^ These plans are a government-subsidised annual chronic disease management plan that help to align health care with a patient’s goals and often include referrals to other healthcare services such as physiotherapy, dietician, and psychological services.^12^

Based on the *Guidelines for Preventive Activities in General Practice*, GPs in Australia are encouraged to ask and provide advice about PA in line with current guidelines.^4^ However, previous literature indicates that GPs often do not initiate conversations about PA with patients and may have different methods of approaching this topic.^14–19^ Existing research has demonstrated that for the GPs that do speak about PA, often they approach it through a combination of verbal PA discussions, PA prescriptions, and acquiring a team of other referred healthcare professionals.^20–22^ In this context, exercise prescription refers to a purposeful and goal-oriented fitness plan that includes frequency, intensity, time, and type (FITT) principles.^23^ Previous research has mainly explored the topic of PA in GP–patient consultations using in-depth qualitative interviews, surveys, and observation methodological strategies. However, these studies have methodological limitations and were potentially impacted by concepts such as social desirability and recall bias, which can potentially skew the data collected, analysed, and concluded, further restricting the accuracy of findings.^24–26^ International and Australian studies had used video recordings of GP–patient consultations to reduce this margin of error or influence in the data and had significant success.^27–30^ Yet, PA has not been explored in the Australian general practice, despite PA being a crucial component to patient health and wellbeing. This study aims to explore (a) how GPs approach PA discussions with their patients and (b) what type of PA is discussed in real-world GP–patient video consultations.

## Method

### Study design

This was a qualitative research project that utilised descriptive content analysis of secondary data of recorded real‐world primary care GP–patient consultations.^31,32^ This approach provides descriptions of experiences while recognising the subjectivity of the diverse consultations in an inductive manner.^31^ The study is reported in accordance with the Standards for Reporting Qualitative Research (SRQR) checklist.^33^ The study’s multidisciplinary research team included early to mid-career clinical and non-clinical researchers with expertise and extensive experience in qualitative primary care research, and a practising physiotherapist and clinical GP.

### Data collection: video recordings

The Digital Library^34^, held with the National Centre for Healthy Ageing at Monash University in Australia,^35^ is a digitised repository containing real-world video‐recordings of health and social care consultations from community, outpatient, and residential care settings. Data in the Digital Library is derived from different parts of the healthcare system, including community health and primary care, hospitals and other acute settings, aged care facilities, telehealth, and outreach services. The Digital Library repository provides an infrastructure for research and education purposes to improve healthcare interactions, communication strategies, improve patient safety, support clinicians, and increase patient health outcomes and consumer satisfaction. This study focused on the general practice context. We used a video collection containing real‐world GP–patient consultations and related data, including transcripts, patient survey logs, and participant demographic data. At the time of this study, the Digital Library held 43 GP–patient consultations, which were used as data for this project that had been collected between August 2021 and February 2022. We used this data repository for our analysis.

GPs with teaching and/or training responsibilities were recruited from GP practices across Melbourne, Australia. They were identified from existing databases of GPs interested in research, public profiles, or previous GP participants (snowballing strategy). GPs were sent an explanatory sheet and consent form about the project, and followed up twice as per the Dillman method of recruitment.^36^ Informed consent was obtained from volunteer GPs before the first recording day. GPs received an honorarium of $120 (approximately 57.75 GBP) per day in recognition of their time.

A research assistant attended each clinic recording day. They explained the study to all patients seeing the participating GP that day and sought informed consent. Patients who did not consent did not have their consultation recorded. GPs and patients were not aware that PA was the topic of research at the time of recording. Video recordings were transferred to a secure hard drive at Monash University with restricted access. These videos were transcribed verbatim using transcription software^37^ for analysis. All identifiers were removed from the video and survey data before analysis.

### Data analysis

**Figure 1.**
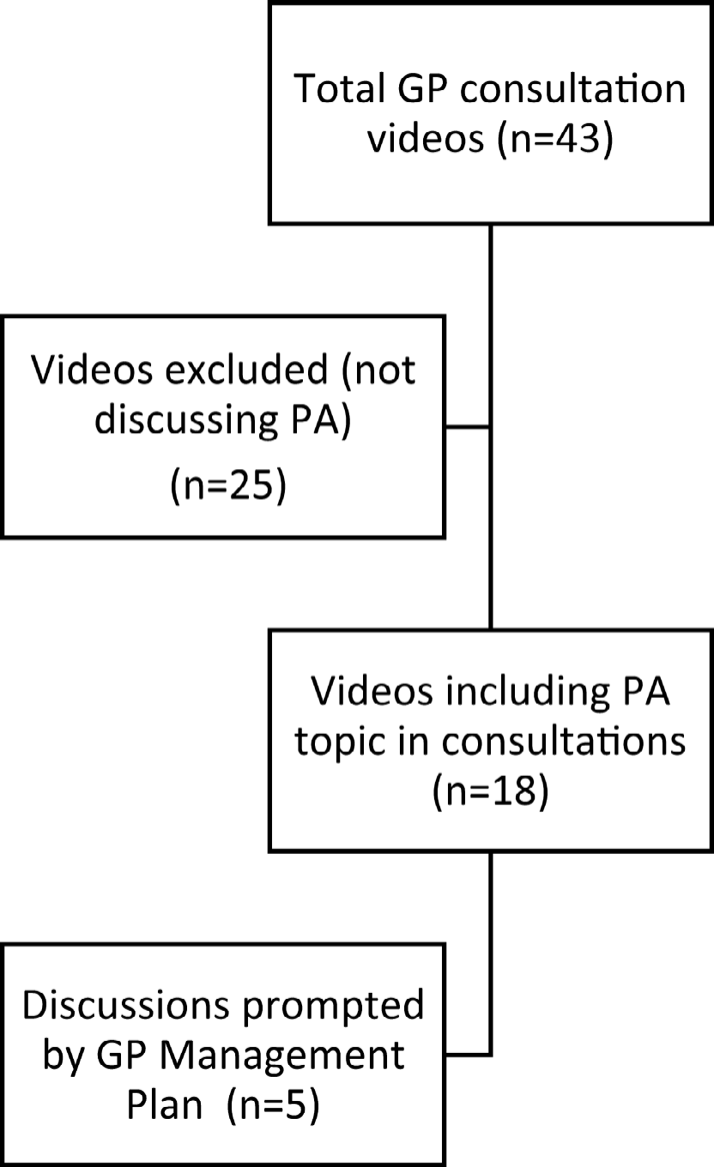
Flow diagram outlining the total number of GP consultations and the consultations filtered at each stage of the analysis process. PA = physical activity

Two researchers (NG and KN) watched all 43 naturally occurring consultations to identify which recordings included some form of PA mention or discussion. Eighteen (41.9%) consultations included the topic of physical activity (Figure 1). NG and KN analysed all 18 consultations looking for (a) how GPs raised the topic of PA, and (b) what type of PA was discussed. Analysis was guided by descriptive content analysis.^31,32^

Each video consultation was viewed and initial notes were recorded in an Excel spreadsheet. The researchers read and re-read the transcripts of each recording to familiarise themselves with the data, and utilised the demographic information stored with the video for additional context when analysing each consultation. Using analysis software (ATLAS.ti), passages of text that included the topic of any form of PA were manually highlighted by researchers (NG and KN) and labelled into codes that were (a) relevant to how PA was discussed by the GP or (b) related to a specific type of PA discussed. These preliminary codes were discussed with the wider research team (NG, KN, and ES) for rigorous debate and discussion. These codes were finalised by the research team, which consisted of early to mid-career researchers, a practising GP and allied healthcare workers, primary care researchers, and qualitative experts. While data saturation is positioned as subjective and situated,^38^ this analysis continued until all the researchers agreed that no new codes were identifiable in the data post‐wider team meeting analysis.

## Results

The majority of patients were aged >45 years (*n* = 16) and female (*n* = 11) (Table 1). The participating GPs in the study were all aged ≥45 years, with >20 years of practice experience (Table 2).

**Table 1.** Demographic information of 18 patients’ consultations that included reference to physical activity (2021–2022)

Patient #	Age range, years	Sex
P01	45–54	Female
P02	45–54	Male
P03	55–64	Male
P04	55–64	Male
P05	55–64	Male
P06	45–54	Female
P07	35–44	Female
P08	55–64	Male
P09	45–54	Female
P10	55–64	Male
P11	55–64	Female
P12	75–84	Female
P13	>85	Male
P14	>85	Female
P15	75–84	Female
P16	25–34	Female
P17	75–84	Female
P18	65–74	Female

**Table 2.** GP demographic information

GP #	Age range, years	Sex	Years in role
GP 01	45–55 years	Female	>20
GP 02	>55 years	Male	>20
GP 03	45-55 years	Female	>20
GP 04	>55 years	Male	>20

### PA discussions varied in approaches and types

Some GPs provided exercise prescriptions when navigating PA discussions that included consideration of patient specific needs. Exercise prescription is a type of PA that includes tailoring personalised exercise advice alongside the patient to meet the patient’s needs. One GP used exercise prescription and worked with the patient to set a PA walking goal that enabled patient autonomy in their PA engagement:

GP 01: *‘Can we try some walks? I want you to do what’s reasonable.’*

P 04: *‘Yeah I-I try to get out and do few blocks around*.’

GP 01: *‘Yeah? so can we do a daily — or how many days a week? What do you think you can do? Let’s start with two or three days walks, three days per week around the block kind of thing?’*

P 04: *‘Yeah*.’

GP 01: *‘Is that kind of 10 minutes? 20 minutes?’*

P 04: *‘Yeah. It’ll probably take me about 20 minutes slowly.’*

GP 01: *‘20 minutes and build up*.’

(Patient: male, 55–64 years. GP: female, aged 45–55 years, 20 years’ experience.)

Another GP tailored exercise to what the patient enjoys and has physical ability to engage with. The health goals of this patient were to keep active and eat healthy, which the GP encouraged and guided:

GP 03: *‘At 77 you can expect to have a little bit more weight, as we get older, and as you can move around well, and you’re generally paying attention to those sort of things. That’s fine*.’

P 17: *‘Yes. Of course. yeah.’*

GP 03: *‘Yeah, so — keep as best as you can keep moving, keep gardening, keep doing what you do. And just pay attention to trying to get five vegetables a day*.’

(Patient: female, aged 75–84 years. GP: female, aged 45–55 years, 20 years’ experience.)

Some GPs approached exercise through the type of generalised advice to promote overall health and wellbeing. Generalised PA can aid to increased mobility and strength, increasing overall wellbeing. One patient wanted to alleviate some muscular discomfort from a fracture injury. Here, the GP utilised the opportunity to educate and promote the engagement with PA to alleviate the patient’s discomfort:

GP 01: *‘And the likelihood is with some stretches and exercises — because also being in that cast for so long, your muscles will have gone a bit weak and now they’ve got to get themselves moving again and strengthen again and I imagine it should just — we should be able to get it to settle.’*

(In consultation with Patient 06, female, 45–54 years. GP: female, aged 45–55 years, 20 years’ experience.)

Five GPs had patients who were engaged with a GP management plan. This plan includes the topic of PA, which GPs used as a tool to raise the topic of PA in a structured and systematic way. One GP example is:

GP 01: ’*Now let me have a look. What have I got on my list — um — I wanted to review our GP management plan overall to see how everything was going*.’

P 07: *‘Yep.’*

GP 01: *‘And exercise wise, or I suppose activity wise?’*

(Patient: female, aged 35–44 years. GP: female, aged 45–55 years, 20 years’ experience.)

At times, consultations involved GPs promoting PA in conjunction with other healthcare referrals. For example, one GP took an educational and PA-promoting approach. Here the GP positions PA as beneficial when used with medications and physiotherapy to improve patient health outcomes:

GP 01: *‘What really works is exercise-based physio. The anti-inflammatories can help a bit if you can tolerate them. Y’know, Nurofen, Ibuprofen that kind of thing, just to settle it down*.’

P 05: ’*I’ve got Ibuprofen.’*

GP 01*: ’Yeah, so taking some of those and seeing a good physio, is what I’ll be suggesting.’*

(Patient: male, 55–64 years. GP: female, aged 45–55 years, 20 years’ experience.)

GPs tailored the type of exercise advice by catering their discussions to suit each patient’s abilities and lifestyle. For example, one GP utilised the opportunity to tailor PA discussions based on the patient’s individual lifestyle factors of being a small business owner with limited time outside of work for PA. The GP also engaged in goal-setting in combination with shared decision making to establish a realistic exercise regimen to recommend:

GP 01: *‘Sometimes even just keeping those steps going, if possible, even if you’ve got a work day like today.’*

P12: *‘Yes.’*

GP 01: *‘Still trying to get to the 5000 or even — do you think you could even aim for five and a half thousand or 6000?’*

(Patient: female, 75–84 years. GP: female, aged 45–55 years, 20 years’ experience.)

When reviewing exercise engagement with one patient, the GP supported their decision for an aerobic activity (running) and advised that it was a positive exercise option for the patient’s specific health goals:

P 02: *‘I probably need to find out what the optimum running per week, probably not every day I would have thought*.’

GP 02: *‘Fair enough. You work out what’s best for you. But it’s great. You’re feeling fit and wanting to really go make yourself healthier by doing that*.’

P 02: *‘Yeah, yeah, definitely. Cardio wise. I’m sure I’m getting healthier from that*.’

(Patient: male, 45–54 years. GP: male, aged >55 years, 20 years’ experience.)

PA was experienced differently by each patient and GPs utilised patient-centred healthcare strategies to align with patients’ changing circumstances, preferences, and expectations. When reviewing a PA goal to walk every day, the patient indicated the change of circumstances and health level. Here the GP adapted their advice to tailor to this change of circumstances and focused on the positive collaborative (physiotherapy) benefits of consistent engagement with health enhancing PA, regardless of the intensity or frequency reached:

GP 01: *‘Are you still getting out for a walk every day?’*

P 04: *‘I just haven’t had energy. I’ve done a little bit of increased, the thing and with the physio now I’m now doing four hours of physio a week*.’

GP 01: *‘Oh good! And that should be able to continue through lockdown, shouldn’t it?’*

P 04: *‘That’s what I’m hoping, yeah. That’s what I’ve gotta ring* [location name] *about*.’

GP 01: *‘Yes. So that’s what I was thinking, because we want to increase with three hours of stuff a week?’*

(Patient: male, aged 55–64 years. GP: female, aged 45–55 years, 20 years’ experience.)

PA engagement was often discussed in relation to other health topics. One GP was discussing the overall benefits of healthy lifestyle modifications and took the opportunity to recommend aerobic-based walking for one patient when discussing weight-related health issues:

GP 01: *‘How does that sound? And in the meantime, get back to walking, and your diet by the sounds of it slowly, slowly we’re getting somewhere. Yeah?’*

(In consultation with Patient 03, male aged 55–64 years. GP: female, aged 45–55 years, 20 years’ experience.)

Some GPs discussed PA by enquiring about different exercise types that patients had indicated they enjoyed. For example, one GP specifically enquired about aerobic exercise (that is, swimming) for a patient who had undergone a shoulder injury to assist in range of motion, pain, and mobility management:

P 01: *‘I’ve been — I’ve been — the last couple of days we’ve been swimming. So, it’s improved a bit more. I found that the swimming is helping*.’

GP 01: *‘Okay, good. So you’re back in the pool?’*

(Patient: female, aged 45–54 years. GP: female, aged 45–55 years, 20 years’ experience.)

Another GP tailored the PA discussion by conducting an assessment and treatment based on using a sit-to-stand functional exercise type used in clinical practice. The GP monitored the over 85-year-old patient’s physical ability during the functional assessment to ensure it was appropriate and safe to perform in a non-supervised environment:

GP 01: *‘So how are you getting up out of the chair? Show me*.’

[Patient shows GP how they get up from chair]

GP 01: ’*Alright. Sit down again. Are you able to get up if you put your hands like this?’*

P 13: *‘Oh no. Oh-oh.’*

[Patient gets up from chair without using hands]

GP 01: *‘Well done. You’ve done it. I want you to practise that. Okay?’*

P 13: *‘Pardon?’*

GP 01: *‘I want you to practise that. Okay. So instead of getting up with the chair that’s what I do with — that’s one of the exercises I want you to do.’*

(Patient: male patient, aged >85 years. GP: female, aged 45–55 years, 20 years’ experience.)

## Discussion

### Summary

This study explored how PA is approached and the type of PA that was discussed in real-world GP–patient video consultations. PA was approached through the use of exercise prescription and general advice, with a noticeable emphasis on aerobic-based exercises, aligning with the WHO PA guideline recommendations.^6^ GPs often included referrals to relevant allied healthcare professionals relative to patient health needs. In five consultations, GPs utilised the GP management plan to prompt PA discussions. GPs adopted a patient-centred approach during most consultations to tailor specific treatment and recommendations to each patient’s needs and abilities. These findings have implications for the ongoing training and development of health promotion in approaching and discussing the topic of PA in routine care for current and future practising GPs.

### Strengths and limitations

The study sample provided real-life consultations of experienced GPs in routine care, contributing to the limited knowledge of how PA is approached in GP–patient consultations. While this study is qualitative, and therefore findings are unable to be generalised, these findings provide valuable insights into GP–patient consultations in a way that minimises social desirability and recall bias as no participants were aware that PA was of research interest at the time of recording, which is a key strength.

A potential limitation of this study was that these consultations are only from one GP–patient consultation in isolation, which limits the understanding of PA over extended periods of time. However, the multidisciplinary research team approach to analysis enabled strong reliability of the interactions at this ‘snapshot’ of therapeutic care. Notably, these consultations were recorded at the height of the Melbourne, Australia COVID-19 restrictions, which may have impacted the priority of health topic conversations at this time. An additional limitation is that GPs consented and volunteered to participate before consultations were recorded, which may have introduced a bias towards favourable ‘good practice’ behaviours in general (even though they were unaware PA was the topic of exploration). Previous literature has highlighted concerns about the ’Hawthorne effect’^39^ in video research. These concerns are oriented with the potential for altered behaviour by participants owing to the awareness that they were being recorded. However, a 2017 US primary care study^39^ found little Hawthorne effect from an in‐person observer and another^40^ audio‐recorded study identified there to be no significant Hawthorne effect on doctor–patient communication, confirming the valuable contribution of real‐life recordings for empirical research. As there were four GPs with multiple recordings in this repository at the time of this study, including a larger scope of patients and GPs with more diverse sociocultural backgrounds would be beneficial.

### Comparison with existing literature

GPs incorporated PA into their health consultations using either exercise prescription, general advice, or a combination of both. Often, the topic of PA was approached using the FITT principles and focused on specific components of frequency and type of exercise, supported by international literature.^22,23,41,42^ The recommendation variations were highly dependent on the patient’s presenting condition. For example, general advice of stretching was provided to a patient post-cast removal in the context of fracture recovery (Patient 6). While the actual advice varied in different ways to PA guidelines and aligning with all the FITT principles as previous literature suggests, this study found that even though GPs did not mention all FITT principles, the advice given was beneficial for optimal health outcomes per specific patient needs.

PA has been demonstrated to enhance overall health and wellbeing for patients.^6^ GPs are in a prime position to promote, educate, and encourage patients about the benefits of engaging with PA however they can.^4,43,44^ Our findings offer support that GPs are including PA in their discussions where appropriate and utilise both opportunistic and systematic approaches to discussing PA. Five consultations had patients with chronic conditions and GPs utilised the GP management plan to include the topic of PA in their discussion, regardless of which chronic condition (or multiple conditions) the patient was living with. The prompting of PA questions in the GP management plan provided the structured opportunity to discuss maintenance and new goal creation to integrate PA to manage patients’ chronic conditions. While the GP management tool is specific to Australia, these findings are relevant to similar general practice care plans internationally, including the UK. International, and specifically UK, PA guidelines^1,5^ follow similar processes and are a positive tool to use for PA discussions with patients. Outside of the GP management plan, GPs educated and promoted PA engagement to their patients to enhance health, which aligns with both Australian and international best-practice guidelines.^1,4,5^

The type of PA discussed varied, however, participating GPs demonstrated a patient-centred approach towards PA discussions, which aligns with best-practice guidelines.^4,45^ A patient-centred approach recognises the personal circumstances of each patient and allows collective support to meet one’s individual needs while having mutual respect and trust from the GP–patient relationship.^44,46^ In particular, GPs respected patients’ autonomy through shared and informed decision making while placing the patients’ needs at the forefront of the care process through considerations of limitations and physical abilities when tailoring the type of PA. GPs demonstrated skillful patient-centred care by offering a suggestion rather than directives when discussing PA. For example, one GP tailored functional movement advice for a patient who was aged >85 years while another recommended aerobic exercise for a patient who wanted to increase cardiovascular health. Findings from this study offer further support for other literature conducted that emphasise GPs displaying a patient-centred approach through mutual understanding, building a therapeutic relationship that establishes trust, fostering collaborative care, and shared decision making to improve patient outcomes.^47–49^ Further, individually tailoring care to each patient helps to promote patient goals and preferences, which builds rapport among GPs and patients, which is crucial for the therapeutic relationship.

### Implications for research and practice

It is important that GPs are well equipped to have conversations that include the topic of PA. This study demonstrates that GPs are including PA discussions in their consultations whether in opportunistic or systematic ways. This study sheds light on how PA is really talked about in GP–patient conversations and can be used as a baseline foundation for improvements to PA conversation strategies in the future. The findings of this study can contribute to establishing teaching and training resources to support GPs in their roles about different ways to talk about PA with patients (for example, opportunistic, or systematic with tools such as the GP management plan). Future development of any PA discussion tools or strategies for GPs should consider co-designing these with patient representatives and GPs to ensure they will be appropriate and maximise GP support and patient health outcomes related to PA. Future studies could explore why more than half of the consultations in this repository did not include the topic of PA and any missed opportunities in this context and could explore how PA discussions improve health outcomes over longer periods of time. It will also help to determine how, and if, PA is followed-up to further understand the effectiveness of these discussions in practice and promoting health management and prevention in routine care.

## Data

It would not be appropriate to publish the data on which the conclusions of this article are based, as this would breach participant confidentiality. The data are real-world video recordings and include patient and clinician identifiable information.
